# Invasive *Saprochaete* Infections: An Emerging Threat to Immunocompromised Patients

**DOI:** 10.3390/pathogens9110922

**Published:** 2020-11-07

**Authors:** Said El Zein, Joya-Rita Hindy, Souha S. Kanj

**Affiliations:** 1 Internal Medicine Department, Wayne State University/Detroit Medical Center, Detroit, MI 48201, USA; gr6712@wayne.edu; 2 Division of Infectious Diseases, Internal Medicine Department, American University of Beirut Medical Center, Beirut 1107 2020, Lebanon; Joyahindy@gmail.com

**Keywords:** Saprochaete clavata, Saprochaete capitata, Geotrichum clavatum, Geotrichum capitatum, Magnusiomyces capitatus, Blastoschizomyces capitatus

## Abstract

*Saprochaete clavata* and *Saprochaete capitata* are emerging fungal pathogens that are responsible for life threatening infections in immunocompromised patients, particularly in the setting of profound neutropenia. They have been associated with multiple hospital outbreaks mainly in Europe. In this article, we present a comprehensive review of the epidemiology, clinical presentation, diagnosis, antifungal susceptibility and treatment of these organisms. The diagnosis of invasive *Saprochaete* disease is challenging and relies primarily on the isolation of the fungi from blood or tissue samples. Both species are frequently misidentified as they are identical macroscopically and microscopically. Internal transcribed spacer sequencing and matrix-assisted laser desorption ionization-time of flight mass spectrometry are useful tools for the differentiation of these fungi to a species level. *Saprochaete* spp. are intrinsically resistant to echinocandins and highly resistant to fluconazole. Current literature suggests the use of an amphotericin B formulation with or without flucytosine for the initial treatment of these infections. Treatment with extended spectrum azoles might be promising based on in vitro minimum inhibitory concentration values and results from case reports and case series. Source control and recovery of the immune system are crucial for successful therapy.

## 1. Introduction

Invasive fungal infections (IFIs) constitute a major cause of mortality and morbidity especially in severely immunocompromised patients. These infections are being increasingly recognized worldwide, partly due to advancements in diagnostic testing, implementation of aggressive chemotherapeutic protocols and use of antifungal prophylaxis in immunocompromised patients. In one report, the incidence of IFIs increased from 23.2 cases / 100,000 patients in 2008 to 31.8 cases / 100,000 patients in 2015 [1]. To date, *Aspergillus*, *Candida*, *Cryptococcus* and *Pneumocystis* remain the most predominant fungal pathogens affecting immunocompromised patients [2]; however, over the past decade, we witnessed the emergence of less common fungal species as causative agents for life-threatening IFIs such as *Saprochaetes* and *Trichosporon* spp. [3]. These fungal organisms can cause blood stream infections as well as invasive and disseminated multiorgan disease.

Saprochaete clavata (formerly Geotrichum clavatum) and Saprochaete capitata (formerly Geotrichum capitatum, Blastoschizomyces capitatus, Magnusiomyces capitatus) are closely related organisms that are often misidentified due to their close phenotypical resemblances [3,4]. They are arthroconidial yeast-like filamentous fungi whose taxonomy has undergone multiple revisions over the years, largely due to changes in the rules of fungal nomenclature [5]. They are microbiologically and phylogenetically related to ascomycetous yeasts and are classified in the family Dipodascaceae, order Saccharomycetales [4,6]. 

The diagnosis of *Saprochaete* invasive infections remains challenging and relies primarily on clinical suspicion and isolation of these organisms from blood, tissue samples or sterile body fluids. Despite advances in diagnostic microbiology, *S. clavata* and *S. capitata* can often be misidentified even in laboratories that are equipped with advanced diagnostic tools such as automated identification systems, internal transcribed spacer (ITS) sequencing or matrix-assisted laser desorption ionization-time of flight mass spectrometry (MALDI-TOF MS) [4,5]. Both organisms appear to be predominantly resistant to echinocandins and fluconazole [7]. Reporting mortality from IFIs can be challenging as infected patients tend to be critically ill and death can often be attributed to numerous factors. Nevertheless, forty-two-day all-cause mortality for both *Mucorales* and *Aspergillus* spp. has been estimated at 28% in one study, compared to 20.5% for *Candida* [1]. Despite early isolation and initiation of adequate antifungal therapy, all-cause mortality rates as high as 40%-80% for invasive *Saprochaete* spp. infections have been reported in some patient groups (Table 1) [8,9,10].

The epidemiology, clinical presentation, diagnosis, antifungal susceptibility and treatment of these organisms will be reviewed in this article. 

## 2. Epidemiology 

*S. capitata* and *S. clavata* have emerged as causative agents responsible for multiple hospital outbreaks predominantly in Europe (Table 1) [3,9,11], with Italy having the highest number of reported cases to date. It is worth noting that outbreaks appear to be more peculiar to *S. clavata* compared to *S. capitata*, with the latter being associated with a high number of sporadic cases reported in several countries around the world such as Brazil [12], India [13,14], Iraq [15], Japan [16], Tunisia [17] and Turkey [18,19]. A recent cohort study in patients with cancer reported a rate of *Saprochaete* spp. infection approaching 3.4 per 100,000 patient admissions [20].

*Saprochaete* spp. can be found in nature, especially in soil [14,21]. They have also been isolated from dairy products [22], as well as dishwashers in household and healthcare facilities [23,24,25,26], making these contaminated sources potential suspects for some of the reported outbreaks. The majority of patients who develop invasive *S. capitata* or *S. clavata* infection are initially colonized with the organism [11]. The site of entry of these pathogens into the human host remains unclear; however, invasion through the respiratory or gastrointestinal tract is likely [3,15]. Damage to the gastrointestinal mucosal barrier and ulcerations induced by chemotherapy might allow fungal translocation, increasing the risk of fungemia [3]. The mode of transmission is not defined; however, the fact that isolates from the same clade were responsible for hospital outbreaks in France suggests that human–human infection in hospitalized patients is possible and could be related to environmental contamination or device colonization [5,9,26]. For instance, a recent French *S. clavata* outbreak was linked to a dishwasher with a deficient heating system [26]. Fly-to-human transmission is unlikely; however, *S. clavata* has been isolated from Drosophila fly body surfaces [2,12,14]. 

The majority of patients with invasive *Saprochaete* infections have, at the time of fungemia, an impaired immunity due to profound neutropenia secondary to chemotherapy administration in the setting of hematological malignancy. There appears to be a particular association with cytarabine use and acute leukemia (Table 1) [3,10,27,28,29,30,31,32]. Although exceedingly rare, invasive *Saprochaete* infections have been reported in solid organ transplant recipients receiving immunosuppressive therapy [33,34,35], and critically ill patients with prolonged intensive care unit stay without underlying malignancy [36,37]. Other underlying risk factors in immunocompromised patients include dysbiosis due to the prophylactic use of antibiotics and antifungal agents in patients prior to the infection [11,16,38]. A case of *S. capitata* infection has also been reported in a patient with CARD9 deficiency—a genetic immune disorder characterized by susceptibility to fungal infections [15]. *Saprochaete* pulmonary infections have been reported in immunocompetent patients with underlying lung disease such as asthma, chronic obstructive lung disease, history of tuberculosis infection or bacterial pneumonia [39,40,41,42,43].

## 3. Clinical Presentation

Disseminated disease is common with *Saprochaetes* especially in severely immunosuppressed patients. Clinical presentation mimics that of invasive candidiasis, and fungemia is common, although *Saprochaetes* tend to affect the lungs and deep organs more frequently than *Candida* spp. [54,60]. Patients often present with an acute febrile illness, which could be the only initial manifestation, or could be accompanied by various other symptoms depending on the sites of disease involvement. Symptoms usually progress rapidly to multiorgan failure, shock and death in the majority of patients despite adequate antifungal therapy [11]. Mortality approaches 60–80%; however, rapid molecular identification and prompt initiation of appropriate antifungal therapy have been shown to decrease the numbers of disease-related deaths [3,9,45,61]. The clinical presentation in immunocompetent patients is less severe, and in this population, S. *capitata* has been exclusively isolated from sputum samples [42]. 

**Figure 1:**
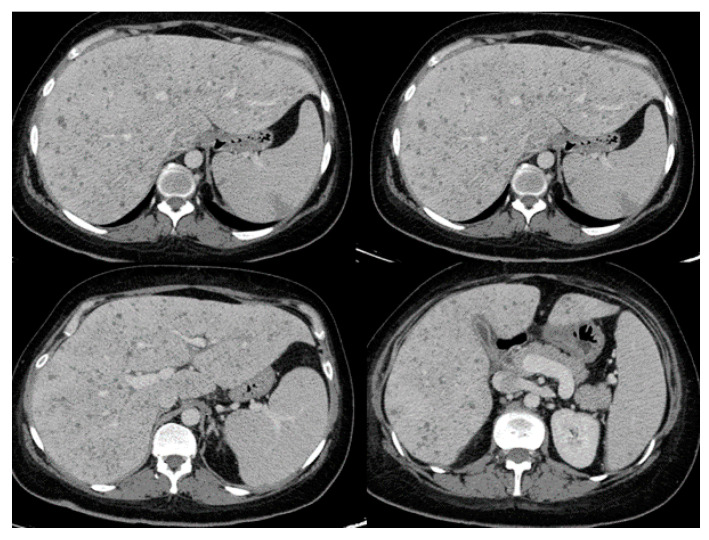
Abdominal computed tomography showing hepatosplenic abscesses in a patient with invasive *Saprochaete clavata* infection (reproduced from Del Principe, M.I et al. Mycoses 2016, 59, 594–601, doi:10.1111/myc.12508 with permission from John Wiley and Sons under license number 4915460075071).

Table 2 lists common presenting symptoms and corresponding radiographic findings as reported in patients with invasive *Saprochaete* spp. infections depending on the site of disease involvement. Patients with pulmonary disease present with shortness of breath and cough which could be productive of purulent or bloody sputum. Most common radiographic findings include diffuse bilateral infiltrates, ground glass opacities, pleural effusion and parenchymal nodules. *S. Clavata* empyema has also been reported, highlighting the importance of obtaining prompt diagnostic thoracentesis and source control [50]. Clinical progression to respiratory failure is common, often requiring intubation and mechanical ventilation. Patients with intraabdominal disease can present with diarrhea, abdominal pain and jaundice in the setting of biliary duct obstruction. Dysuria and hematuria can be present in cases of renal and bladder involvement. Rarely, *Saprochaetes* can cause peritonitis and abdominal compartment syndrome [45,52]. The most common radiographic findings include hepatosplenomegaly and hypodense parenchymal lesions or nodules involving deep organs (Figure 1). Central nervous system (CNS) involvement can manifest as high-grade fevers, mental status changes and seizures that can progress to status epilepticus. Even if the patients recover from their illness and respond to antifungal therapy, they are at risk of developing long term neurological sequalae [10]. A brain mass or multiple lesions with or without surrounding edema and hemorrhages are usually seen on imaging. Skin lesions are seen in some of the patients with disseminated disease. Reported cutaneous manifestations include erythematous nodules and papules on the lower extremities and back mimicking lesions seen in disseminated candidiasis [62]. Black necrotic plaques involving the perianal area, and wound infection at the site of a laparotomy scar have also been reported [33].

## 4. Diagnosis

The diagnosis of invasive *Saprochaete* spp. infection is proven by histopathologic or direct microscopic visualization of the fungi from a normally sterile body site [63]. The clinical significance of isolating these organisms from respiratory, urinary or stool cultures of immunosuppressed patients remains unclear; however, in the absence of other identifiable organisms and in the setting of a clinically documented infection, this represents probable invasive fungal disease [9,54]. In the absence of host factors such as immunosuppression or prolonged neutropenia, the isolation of *Saprochaetes* from non-sterile sites likely represents a contamination and care must be exerted when interpreting such microbiological results [14,42].

**Figure 2:**
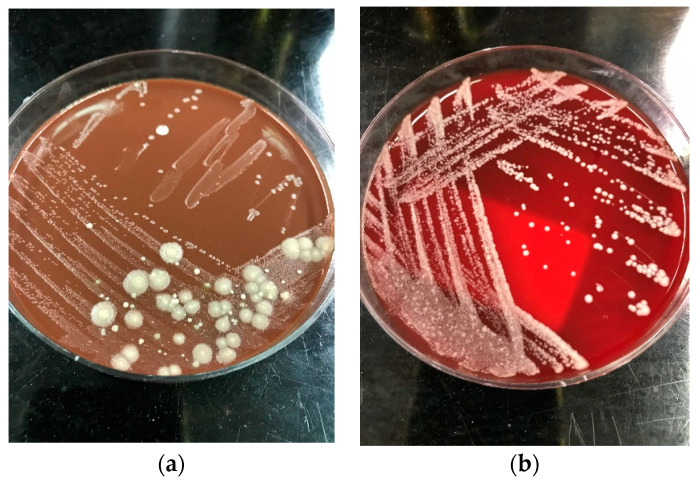
Dry cottony colonies with frosted glass appearance of *Saprochaete* spp. on (**a**) blood agar and (**b**) chocolate agar, respectively. The isolates were incubated at 26 °C for 72 h and later identified as *S. capitata* by MALDI-TOF.

**Figure 3:**
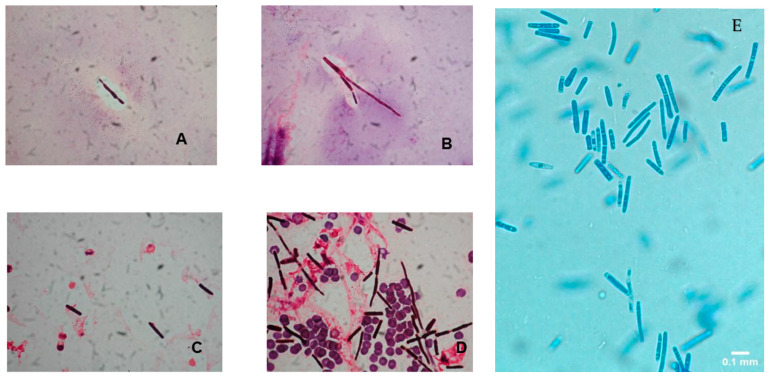
(**A–D**) Gram stain preparation of blood cultures showing arthroconidia and hyphal elements of *Saprochaete clavata*. Magnification ×1000 (reproduced from Del Principe, M.I et al. Mycoses 2016, 59, 594–601, doi:10.1111/myc.12508 with permission from John Wiley and Sons under license number 4915460075071). (**E**) Lactophenol cotton blue preparation of peritoneal fluid cultures showing arthroconidia suggestive of *Saprochaete* spp. infection. The organism was identified as *S. capitata* by MALDI-TOF. Magnification ×40.

The colonies of *Saprochaete* spp. grow on a multitude of fungal media within 24–48 hours; however, cultures may require incubation for up to 5 days. *S. clavata* and *S. capitata* are often misidentified as they are identical in macroscopic and microscopic analysis [4,28]. This misidentification may have contributed to the significantly lower numbers of reported *S. clavata* infections prior to the availability of more advanced diagnostic modalities [3,11]. Distinguishing between these 2 organisms to the species level is important for epidemiological and outbreak investigation purposes, as well as for clinical reasons, as *S. clavata* and *S. capitata* can have different antifungal susceptibility profiles [7]. Macroscopically, *Saprochaete* spp. form yeast-like, farinose, dry cottony colonies with frosted glass appearance on the plate (Figure 2) [3,30]. Microscopically, they form true fragmented hyphae, pseudo-hyphae, arthroconidia, annelloconidia and blastoconidia [3,42,44] (Figure 3). 

*Saprochaete* spp. are urease negative, a characteristic that helps distinguish them from *Trichosporon* spp. [28]. *S. clavata,* unlike *S. capitata,* has the ability to assimilate carbon sources such as cellobiose, salicin and arbutin [5,12]; however, many *S. capitata* strains could grow on all carbon sources [5], and as many as 15% of *S. clavata* strains do not assimilate cellobiose, making biochemical testing alone unreliable for accurate species identification (Table 1) [11]. Moreover, commercial test kits often lack salicin and arbutin [4,64]. Both species are thermotolerant with optimal growth observed at temperatures between 30 °C and 40 °C [4,5]. In one study, *S. clavata*, unlike *S. capitata* isolates, did not grow at temperatures above 45 °C, and isolates preincubated at that temperature became nonviable and demonstrated no regrowth [5]. Differentiation between both species based on incubation temperatures, however, is unreliable as other studies demonstrated that both species exhibited similar growth patterns at different temperatures (Table 1) [23,26].

Commercial systems for yeast identification such as VITEK-2 (ID-YEST card; BioMérieux) and API ID32C (BioMérieux) can successfully identify some of the *S. capitata* isolates; however, none of the systems cover *S. clavata* [3,11,30,35,48]. Nucleotide sequencing of the ITS and partial large subunit (LSU) loci can discriminate between *Saprochaetes* at the species level; however, misidentification is common as the diagnostic ability of these tests is dependent on the accuracy and public availability of the nucleotide sequences (e.g., GenBank) [4,5]. Multilocus sequencing of protein binding loci such as *Rbp2*, *Act* or *Tef1α* might allow for more accurate identification [5,11]. MALDI-TOF mass spectrometry is a useful and reliable modality for the identification and discrimination between *Saprochaete* spp. and other arthroconidial fungi; however, as with nucleotide sequencing, the accuracy of this test is highly limited by the quality and number of mass spectra available in commercial and in-house fungal libraries [11,65]. *S. capitata* and *S. clavata* are better distinguished by MALDI-TOF when newer databases such as Bruker BioTyper (3.0 or 3.1) (Bruker Daltonics, Bremen, Germany) or expanded in-house databases are used (Table 1) [8]. The development of polymerase chain reaction (PCR) assays that are highly species specific may be useful in early identification of the organisms from blood or tissue samples, allowing prompt initiation of adequate antifungal therapy; however, such assays are not yet available for commercial use [66]. In the setting of a suspected outbreak, whole-genome sequence (WGS) typing is the best method to determine clonality and to infer strain relatedness [3]. A single clone was identified by WGS typing as responsible for most cases of a French cluster of infections [9,67]. Recently, the investigation of an *S. clavata* outbreak in France demonstrated that clinical and environmental isolates were clustered within the same clade and the outbreak ended after discarding the contaminated dishwasher and kitchen utensils [26]

Arthroconidial fungi can cross react with *Aspergillus* galactomannan (GM) and serum 1,3-beta-D-glucan (BDG); however, these tests are not sensitive, nor specific for *Saprochaetes* [3,17,49] and therefore have little clinical utility for the diagnosis of invasive infections with these organisms. It is worth noting, however, that a positive GM test in a profoundly neutropenic patient with clinical findings suggestive of invasive *Candida* infection should prompt physicians to suspect invasive *Saprochaete* disease, especially that these organisms are resistant to echinocandins and fluconazole, which are the drugs of choice used for the treatment of invasive candidiasis [49].

## 5. Antifungal Susceptibility and Treatment

No clinical breakpoints are defined for *Saprochaetes*; therefore, antifungal susceptibility results should be interpreted with caution. Based on in vitro susceptibility results from numerous case reports and case series, these organisms appear to be intrinsically resistant to echinocandins and highly resistant to fluconazole [3,11]. A mutation in the *FKS* gene hot spot 1 (*FKS HS1)* which codes a subunit in the β-1,3-D -glucan synthase was detected at a position highly associated with echinocandin resistance in *S. capitata* isolates [68]. Prolonged treatment with echinocandins (particularly caspofungin) appears to be a risk factor for breakthrough infections with these organisms [32,68], although breakthrough IFIs while on fluconazole, posaconazole and amphotericin B have also been reported [17,38,45,48,49,52]. Minimum inhibitory concentration (MIC) values for voriconazole (0.03–1 mg/L), amphotericin B (0.03–2 mg/L), itraconazole (0.01–1 mg/L) and posaconazole (0.03–1 mg/L) are generally low for both fungi [7,11,29,34,36,44,45,47]. MIC values for flucytosine are higher for *S. capitata* (0.06–64 mg/L) compared to *S. clavata* isolates (0.25–0.5 mg/L) [7,12,48,50], while MIC values for isavuconazole appear to be high for both species (1– 4 mg/L) suggesting resistance to this antifungal agent [69]. Acquired resistance to amphotericin B and flucytosine during treatment has also been reported [3].

To date, there is no established therapeutic regimen for the treatment of invasive *Saprochaete* spp. infections, largely due to the rarity and challenging diagnosis of these organisms and lack of standardized antifungal breakpoints [3]. All treatment recommendations are based on expert opinion and extrapolated data from case reports and small case series. The list of antifungal regimens and duration of administration reported in the literature to treat *Saprochaete* infections is extensive. In general, echinocandins and fluconazole should be avoided given the high in vitro MIC values and the frequent numbers of breakthrough *Saprochaete* infections reported while patients are receiving these drugs [7]. High-dose fluconazole might be an appropriate treatment when isolates are susceptible [17,28]; however, avoidance of this drug for empiric therapy might be prudent given the high probability of resistance. Mixed results have been reported with the use of voriconazole monotherapy [17,27], a formulation of amphotericin B monotherapy [11,33] or a combination of amphotericin B and voriconazole [11,27,29,38,44] for the treatment of both *S. clavata* and *S. capitata* infections. Combination therapy with caspofungin and voriconazole has also been reported given the potential in vitro synergy, however, with similar mixed results [11,30,70,71]. The European Society of Clinical Microbiology and Infectious Diseases (ESCMID) guidelines published in 2014 recommend any formulation of amphotericin B with or without flucytosine as initial therapy [72]. Antifungal agents are often associated with significant side effects ranging from nephrotoxicity, electrolyte derangements, neurotoxicity, bone marrow suppression and hepatotoxicity among others; therefore, when using combination therapy, clinicians should balance the potential benefits versus risks of adding a second antifungal agent.

The presence of a central venous catheters (CVC) may be a risk factor for the development of catheter-related *Saprochaete* spp. infections. D’Antonio et al. investigated six *S. capitata* strains recovered from patients with CVC-related fungemia. Restriction enzyme DNA analysis of clinical isolates from blood and catheter tips were identical, and all isolates showed high ability to produce slime in glucose-containing solutions [56]. In a clinical setting, this characteristic may allow *Saprochaete* spp. to form biofilms on CVCs and other prosthetic medical devices and may contribute to the pathogenic potential of these organisms [56,73]. Removal of the CVC within 5 days after onset of infection appeared to positively influence outcomes in patients with *S. capitata* catheter-related infection in one study [54]; however, no firm evidence in the form of randomized controlled trials (RCTs), quasi-RCTs or large observational studies exists in the literature to support this practice. Timely administration of antifungal agents and source control (e.g., drainage of an empyema or retroperitoneal fluid collection) are a mainstay for the treatment of invasive *Saprochaete* spp. Infections [3,74,75]. Recovery from immunosuppression likely plays a crucial role in successful treatment [11,31]. The use of granulocyte stimulating factor (G-CSF) or Interferon-gamma in combination with antifungal therapy has been successful in eliminating the infection in some patients [29,76]; however, the impact of these regimens on clinical outcomes remains unclear. 

## 6. Conclusions

A wide array of host and environmental factors may contribute to the increased risk for infection with *Saprochaetes*; however, definite associations are yet to be evaluated. The incidence of these infections may further increase with the development of novel chemotherapeutic and immunosuppressive therapies; therefore, physicians should vigilantly monitor the emergence of such rare pathogens in the hematologic malignancy population. Prophylactic and empirical antifungal protocols should be updated constantly to reflect the changing local epidemiology of IFIs. 

## Figures and Tables

**Table 1:** Epidemiological and clinical features of *S. clavata* and *S. capitata* as reported in the literature.

	*S. clavata*	*S. capitata*
**Country**	Italy [3,27,31,34] Spain [44] China [30] Czech republic [11] France [9,29,45]	Iraq [15] Lebanon [33] Egypt [42] Tunisia [17] India [13,14,35] Turkey [10,18,19,46] Italy [28,36,37,47,48,49,50,51] Brazil [12] Belgium [52] USA [38] Japan [16,53] Spain [22]
**Outbreaks**	Multiple outbreaks in Italy [3,27] Multicenter outbreak in France [9] Outbreak from contaminated dishwasher and utensils in France [26]	Outbreak in Italy in 1984 [51] Outbreak from contaminated milk in Spain [22] Outbreak in the intensive care unit in Japan [53]
**Mortality**	65–80% [8,9]	40–75% [10,28,54,55]
**Risk Factors ^ϐ^**	Profound neutropenia [3,11,27,29,34] Central venous catheter [3,11,29,45] Chemotherapy (predominantly for acute leukemia) [3,8,27,30,32,45] Prolonged use of broad-spectrum antibiotics [3,11,27,30] Prior antifungal use for prophylaxis or treatment [27,34,45] Hematopoietic stem cell transplant [44]	Profound neutropenia [10,12,14,18,49] Central venous catheter [10,16,36,38,46,54,56] Chemotherapy (predominantly for acute leukemia) [8,12,13,17,32,52] Prolonged use of broad-spectrum antibiotics [12,17,33,52] Prior antifungal use for prophylaxis or treatment [10,12,46,52] Hematopoietic stem cell transplant [28,46] Critical illness and prolonged intensive care unit stay [36,37] Gastrointestinal disease (e.g., perforation or biliary stasis) [11,13] Total parenteral nutrition [46] CARD-9 deficiency [15] Immunosuppressive therapy (tacrolimus, mycophenolate mofetil, prednisone) [33,35,54]
**Source of Isolation ^ψ^**	Blood [3,7,11,27,29,30,34,45] Bronchial / sputum sample [27,34] Ascitic fluid [45] Surgical site [34] Urine [11,27] Stool [27,29] Anal mucosa [44]	Blood [7,10,13,14,17,18,28,36,37,38,47,49,52] Central venous catheter tip [10,46,54] Bronchial /sputum sample [7,14,28,33,35,49] Pleural fluid [7,50] Surgical site [33] Urine [19,33] Bile culture [33]
Carbon Assimilation *	(+) cellobiose (+) Salicin (+) arbutin	(−) cellobiose (−) Salicin (−) arbutin
**Growth Temperature**	Optimal growth: 30–40 °C Partial growth at 45 °C. Robust regrowth at lower temperatures ^ф^	Optimal growth: 30–40 °C No growth at temperatures > 45 °C. Isolates become nonviable ^ω^
**MADLI-TOF Platforms Used**	**Successful identification** MALDI-TOF Biotyper system (Bruker Daltonics) [11,27,29] MALDI-TOF MS-Vitek (bioMérieux, Marcy l’Etoile, France) [30] MALDI-TOF SARAMIS (bioMérieux, Marcy l’Etoile, France) [3] **Failed identification** MALDI-TOF MS-Vitek (bioMérieux, Marcy l’Etoile, France) [3]	**Successful identification** MALDI-TOF (Bruker Daltonics) [19,36,37,47,57] MALDI-TOF MS-Vitek (bioMérieux, Marcy l’Etoile, France) [58] **Failed identification** MALDI-TOF Axima-SARAMIS (Shimadzu-AnagnosTec) and MALDI-TOF Biflex III-BioTyper (Bruker Daltonics) [59]

**^ϐ^** Acute leukemia and prior exposure to caspofungin have been shown to be independent risk factors for the development of *Saprochaete* infections [32]. To the best of our knowledge, the remainder of the listed risk factors have not been shown to be independently associated with increased risk of *Saprochaete* infections in a multivariate analysis. ^ψ^ *S. capitata* is more frequently isolated from sputum or bronchial samples compared to *S. clavata.* Both species are isolated from blood samples frequently. * Approximately 15% of *S. clavata* strains do not assimilate cellobiose; some *S. capitata* strains can assimilate all carbon sources. **^ф^** S. *clavata* and *S. capitata* isolates exhibited similar growth at a range of 30–48 °C. No growth seen at temperatures >48 °C [26]. **^ω^** *S. capitata* isolates grew at a range of 5–47 °C in one study [23].

**Table 2:** Common presenting symptoms and corresponding radiographic findings reported in patients with invasive *Saprochaete* infections.

	Presenting Symptoms	Radiographic Findings
**Pulmonary Involvement [17,27,29,30,35,50]**	Fever Respiratory distress Cough with purulent sputum	Pulmonary infiltrates Parenchymal micronodules Ground glass infiltrates Pleural effusion
**Intraabdominal and genitourinary Involvement [15,17,19,27,28,29,30,34,45,52]**	Fever Diarrhea Jaundice Dysuria Hematuria Abdominal compartment syndrome	Hypodense parenchymal lesions that could involve the liver, spleen and/or kidneys Hepatosplenomegaly Biliary duct obstruction Abdominal ascites Abdominal wall collection
**Central Nervous System Involvement [10,27,44]**	Fever Mental status changes Seizures or status epilepticus	Brain mass or lesion(s); Surrounding brain edema and hemorrhagic foci can be present
**Skin Involvement [17,33,44]**	Asymptomatic, blackish-brown necrotic plaques around peri-anal area Erythematous nodules and papules on legs and back Laparotomy wound SSTI	Not applicable
